# Sustainable human resource management the mediating role between work engagement and teamwork performance

**DOI:** 10.1371/journal.pone.0271134

**Published:** 2022-08-04

**Authors:** Virginia Navajas-Romero, Nuria Ceular-Villamandos, Manuel Adolfo Pérez-Priego, Lorena Caridad-López del Río

**Affiliations:** Department of Statistics & Business, University of Cordoba, Cordoba, Spain; Universidad de Murcia, SPAIN

## Abstract

The present work aims to analyze the properties of the working conditions recorded in the Sixth European Working Conditions Survey (EWCS); with it, it has being built seven independent indexes about different aspects of work’ quality in the health sector, and these constructs are used to evaluate their effects on work engagement (WE). In this sense, the originality of incorporating teamwork as a modulating variable is included. To analyze the effects of the job quality index (JQI) on the WE, a logistic regression model is proposed for a total of 3044 workers within the health sector, differentiating between those who work or not in a team; in a first stage and these estimates are compared with those obtained using an artificial neural network model, and both are used for the consideration of the research hypotheses about several causal factor. An important contributions of the study, it is related to how work commitment is mainly influenced by prospects, social environment, intensity and earnings, all of them related to job performance. Therefore, knowledge of the determinants of work commitment and the ability to modulate its effects in teamwork environments is necessary for the development of truly sustainable Human Resources policies.

## 1 Introduction

From an organizational perspective, human resource management (HRM) is a key element to achieve a sustainable competitive advantage in the business environment [1]. Organizations need to sustainably manage relationships with stakeholders [2]. Agents and companies are affected by the behavior and actions of organizations; and in turn, these actions also impact the stability of corporations [3, 4]. During this process, it is a central issue, for the company, to establish competitive advantages based on value creation [2, 5]. The stakeholder theory identifies that, for companies, not only results prevail, but, also, a non negligible amount of managerial attention must be provided to organizational interests; it is central that organizations try to understand and care about the impact that corporate activities have on the different agents involved [6]. It is these groups who expect companies to respond to social needs; and these issues go beyond economic considerations [7]; alto, it is necessary to make agents relate to companies that cover them, avoiding relationships with institutions that do not meet their expectations of social behavior [8–10].

Following this idea, the interest in this problem is reflected in the literature through the proliferation of academic studies on sustainability in different fields of knowledge, such as economics [11], management [12], social responsibility corporate [13, 14], etc. This concern is also materialized from a political perspective through the framework established by the UN in the 17th Sustainable Development G oals (SDGs). Within these objectives we find two lines of action related to the labor market, specifically, objective number eight, which raises the concept of decent work and objective number three, which is related to the promotion of health and occupational well-being of workers [15].

HRM has become a topic of great importance for current academic research [16] since reaching an optimal point of management of socially sustainable and responsible human resources is a critical factor in terms of improving quality of life for employees [17] and this improvement is a decisive factor in the competitive and sustainable differentiation of companies. In the business field, HRM has become a strategic tool for creating business value through sustainable competitive advantages [18]. Also, it plays a key role in the way in which business strategy is designed due to its index implementation at different levels: corporate, competitive and functional. At the corporate level, the HRM generates resources and capacities that are valuable and difficult to imitate [19] at the competitive level, the HRM promotes differentiation strategies [20]. At a functional level, HRM promotes strategies based on innovation [21], quality [22] and transparency [23]. The literature on Corporate Social Responsibility (CSR) analyzes how these policies influence the perception of companies by external interest groups, such as customers, governments, markets in general, and other agents [24, 25]. When organizations apply CSR initiatives, they introduce socially responsible organizational routines within their management, and this originates an improvement of the corporate culture by considering the key role that employees perform in work engagement and organizational success [26, 27], creating value for each stakeholder [28, 29].

In the academic literature, work engagement has been widely analyzed in relation to economic results due to its importance in business development [30, 31], with job satisfaction [32, 33]), job security [34], leadership [35], work community [36], the job enviroment [37]. Following this idea, an organizational factor widely related to performance, engagement and labor participation is teamwork [38]. The literature indicates that aspects such as decision-making, time management, role recognition, integration capacity, interpersonal communication, empathy, responsibility, recognition of leadership and respect are due to teamwork [39]. However, studies that analyze the relationship between work engagement and teamwork are scarce [40], although this link is a key factor for work engagement in an efficient functioning organizational and business performance [41]. Following this idea, teamwork is a key factor in contributing to final results, and therefore to the success of the organization, but its mediating role has not been sufficiently examined; the published studies have been oriented to other sectors, such as the building industries [42], social media [43] or airlines [44].

This research adopts an objective perspective based on HRM in the healthcare sector, which means that the focus is on the main characteristics of the activities that can be observed and that are related to the work engagement of employees through teamwork. However, the internal management and impact of these actions remain relatively unexplored [45]. Organizations can apply various CSR initiatives and, considering the critical role that employees play in organizational performance and success, introduce socially responsible procedures within their management that improve knowledge and corporate culture [26]. Applying social responsibility initiatives, such as promoting fluid relationships between employees and managers or considering the interests of employees, will generate trust within the company [45]. A socially responsible approach to HRM can advance the literature on teamwork by creating more space for social interactions and dialogues between people within organizations that result in improved performance and long-term employee work engagement. term. There are few studies that analyze work sustainability through the mediation of teamwork; in this sense the escarce bibliography on this topic, analyzes sustainability [46, 47] in the health sector without taking into account the influence of teamwork, or if in the health sector teamwork is not related to work engagement [48].

Most European countries have opted for management based on improving the efficiency and performance in managing public health, through accountability and the imposition of excess workloads [49] with results such as burnout [50], low levels of well-being [51] and less work engagement [52]. Specifically, this is the main objective of this document: the analysis with a holistic and systematic approach of the state of HRM in the European health labor market, and how teamwork can produce effects on the work engagement of the employees. Following this idea, this article aims to analyze the need for HRM in the European healthcare market through teamwork activities, which could improve not only the quality of life of its citizens, but also the organizational performance of the healthcare sector. The precise objectives are the following: to measure the relationship between work engagement and HRM in the health sector following the holistic approach and to identify the components of the dimensions of work that most impact on the construction of work engagement through teamwork. This research has identified the dimensions of teamwork that have a greater impact on cooperative work engagement. Under these circumstances, this research is based on understanding the importance of CSR practices in employee management and evaluating the impact that these initiatives have on the legitimacy of the organization [53].

The results could contribute to the analysis of the labor market, business management and public policies in Europe, especially taking into account the human development objectives recently identified by the UN [54]. The implemented model could help human resources professionals in the health sector to propose efficient human resources management strategies that optimize both individual well-being and company performance. This study offers contributions that complement the previous literature; this study extends the previous works in the field of sustainability [55] analyzing the role of teamwork as a determinant of actions. HRM internal practices and the impact that these activities have on employee work engagement, from the perception that healthcare employees have of the internal HRM practices developed by the companies for which they work. Self-reported performance variables are used; this aspect makes the consideration of employee perception especially relevant. The following research question arises from the above objectives: in the health sector, can the European Union, through teamwork, propose a truly sustainable long-term work scenario in terms of work engagement?

## 2 Work engagement and teamwork

The perspective used in this study is based on the generally accepted idea that HRM are actions carried out by the company for the advancement, maintenance or promotion of some social good, beyond the immediate interests of the company and its shareholders and what is required by law [56]. According to Hendry [57], people management aims to promote employee engagement, human resource management and the achievement of business objectives through practices such as promotion, equal opportunities in place work, balance between work and family, ethical training of staff, participation of employees in decision-making, open communication and ethical leadership [58].

Personal engagement at work is defined as a positive attitude related to vigor, dedication, and absorption [59]. The reason that work engagement influences so many work dimensions is because it produces a state of intense concentration in the job’s performance where time passes faster than normal and employees perceive that they have difficulties in departing from work [60]. In recent years, the concept of work engagement, from the organizational perspective, is gaining relevance due to the effect it produces on employees in terms of performance and achievement of objectives [61, 62], productivity [63, 64] and employee retention [65, 66]. Health professionals are subject to a high degree of pressure; this circumstance can affect their professional performance and therefore, the quality of care provided. This sector is characterized by having schedules that include 24-hour shifts, night work, high-stress situations, very rigid action protocols, and the need to make quick decisions that affect treatments and hospital resources [67]. In this scenario, the mental health of health professionals can be affected, presenting anxiety, depression, insomnia, psychological distress, post-traumatic stress and exhaustion [68]. In this sense, work engagement could help deal with this work context. The literature indicates that work commitment has translated into a higher quality of patient care, although it is influenced by contextual factors such as structural empowerment, social support, efficacy and optimism [69]. In addition, work engagement has been revealed as a protective factor against burnout in all its dimensions (emotional fatigue, depersonalization and personal fulfilment) [70] and is related to job satisfaction, because it helps to make facing the physical, emotional, intellectual problems and spiritual load that are produced by caring for the sick [71].

Teamwork coupled with work engagement is a key variable for good organizational and business development [41]. In fact, the literature indicates that the aspects that are promoted through teamwork are work engagement, decision-making, time management, role recognition, integration capacity, interpersonal communication, empathy, responsibility, recognition of leadership and respect for co-workers [72]. Teamwork is a complex, dynamic and multidimensional skill aimed at achieving common goals, which implies the personal willingness to collaborate with others in carrying out information exchange activities, assigning responsibilities, resolving conflicts, and making a contribution. to the improvement and development of the group [73]. From an organizational perspective, the analysis of teamwork is not new [74, 75], but study has focused mainly on the personal characteristics of team members [76], organizational culture [77], administrative support [78], management style [79] and incentive mechanisms [80]. Studies that analyze teamwork and its relationship with work engagement are rare, however, we can find some research in this regard [40, 81]; these focus on this relationship from the point of view of the influence of leadership, or of certain communication methods in committed teamwork.

The key to the success of incorporating teamwork in human resource management is based on the fact that work teams provide a great diversity of skills, knowledge, experiences and attitudes to compete in the dynamic and competitive environments they face [82]). For a work-group to be efficient in terms of task completion and performance, interdependent skills are required, including the generation, promotion, and implementation of ideas [83, 84]. Janssen [85] defines the theory of Innovative Work Behavior (IWB) as the creation, introduction and intentional application of new ideas within a group or work organization, with the aim of benefiting the performance of the work group or the organization. Deepening the management of the work team, the members of a team must be able to quickly develop the correct combination of competencies or skills to achieve the group’s objectives [86]. In fact, the integration of these factors makes it possible to find fast, innovative and flexible solutions, capable of producing great results in the organization. Teamwork allows an increase in group performance through the degree of achievement of collective objectives via efficiency, internal processes and coordination [75].

However, it may happen that there are differences in the development of skills between team members [87], hence each member must be able to influence how they will work on this problem. This issue affects the time horizon of the working group and the rotation of its members [88]. Following this idea, each member of the organization must value the importance of team learning, in this way it will be possible to achieve fast and innovative solutions, capable of promoting sustainable development. In the medium and long term, the collaboration of the work-team will be stronger and of better quality to the extent that each member of the group is able to contribute by providing energy and knowledge to the rest [89]. Following this idea Evans [90] showed that the exchange of knowledge between workers is favored by links between which are trust and the duration of relationships between workers, due to the reciprocity between their members [91]. In fact, the short-term time horizon and high turnover are factors that condition teamwork, since they imply unstable collaborations where the transfer of skills is limited [86].

## 3 Hypothesis

The management of health services represents a subject intensely debated by international and national organizations and society, due to the profound implications it has for citizens. However, despite the importance of the sector for society, the European Union cannot intervene directly or impose binding decisions, because public health is a common task shared among the member countries. The differences between public health systems at the European level are determined by factors such as infrastructure, the quality of public policies, culture, legislation, and human resource management [92]. Going deeper into this idea, there are differences between the technical efficiency (obtaining more products than inputs) and the allocative efficiency (distribution of resources) of each country [93]. Based on this differentiation, in Europe, the health system has adopted the Beveridge model (Cyprus, Denmark, Spain, Finland, Ireland, Italy, Latvia, Malta, Portugal, United Kingdom and Sweden), which is based on a predominantly National Health System, or the Bismarck model (Germany, Austria, Belgium, Bulgaria, Croatia, Slovakia, Slovenia, Estonia, France, Greece, Hungary, Lithuania, Luxembourg, Netherlands, Poland, Czech Republic and Romania), which implies that the financing of the health system is carried out through compulsory contributions to social security, generally through employers and employees, that is a Social Health Insurance System, altough, in the European case, both share some characteristics. However, in addition to these two models, there is also the mixed model, in which private financing of voluntary insurance systems is significant (Private Health Insurance System) [92].

The object of the investigation is therefore to contrast the hypotheses that are presented below:

*H*_1_. Companies that implement HRM in the health sector have a greater probability of increasing their work engagement when developing their work activity in work teams and when they organize activity in work teams, and tend to improve.
*H*_2_. Companies that implement HRM in the health sector have a greater probability of increasing their work engagement when developing their work intensity and their earnings.
*H*_3_. Companies that implement HRM in the health sector have a greater probability of increasing their work engagement when developing their social environment.
*H*_4_. Companies that implement HRM in the health sector have a greater probability of increasing their work engagement when developing their prospects.

## 4 Sample and research methodology

### 4.1 Sample

Health care varies along in Europe. In some countries, patients should enter using the primary care, as a filter to access to specialists, with more or less freedom of choice of doctor, being quite common the co-payment either in primary care, specialized care, hospital admission, laboratory and imaging tests. Doctors are paid for services in some countries, by capitation, or a salary (in hospitals and specialized care), or a mix of these.

The number of doctors per 1000 inhabitants is (in 2017) 3.7, with a range of 6.3 in Greece to 2.4 in Poland, and 14.3 nurses in Finland to 3.3 in Greece; hospital’s beds vary between 8 in Germany to 2.2 in Sweden. The public and private health expenditure as a proportion of Gross Domestic Product oscillate between 11.3 in France and 5.2 in Rumania. In summary, there are quite different systems, but with a clear trend of homogenization within the EU in the results. although with different level of financing and organization. The Health Ministry in Spain produced the study ‘Health care systems in the EU countries’ (2019), with a broad comparison between the continent systems.

This study uses the information about working conditions included in the Sixth European Working Conditions Survey (EWCS). They are supported by the workers themselves, and this leads to elaborate seven independent set of variables about the quality of work [94]. The sample was obtained randomly from the survey database, taking into account the different occupational levels of the active population in each country. Due to its multidisciplinary nature, the EWCS is a tool frequently used in studies and research work on the conditions of workers in Europe [94–96]. This survey is designed to respond to the analysis of these conditions from the prism of seven different dimensions: physical environment, age group, work intensity, prospects and earnings by occupation, quality of free time from work, skills and discretion, social environment and sector [97].

The relationship between work engagement and decent work was analyzed within the health sector, differentiating between workers who do not work in teams and workers who do. The EWCS surveyed a total of 3044 workers, of which 75.3% work as a team (n_1_ = 2,309), compared to 24.0% who do not work as a team (n_2_ = 765).

### 4.2 Variables

The objective or response variable considered is work engagement (WE). This variable has been constructed from five items included in the EWCS questionnaire: (1) In my work I feel full of energy; (2) I am excited about my work; (3) Time flies when I’m working (4) I feel exhausted at the end of the workday and (5) I doubt the importance of my work. Previous research validates and standardizes the use of this survey in studies on engagement at work [98]. The explanatory variables are the seven EWCS dimensions of quality of work conditions listed above. These indices are formed, in turn, by a vast range of items that make up decent work (Table 1). The results can be interpreted as a comprehensive measure of decent employment in general consisting of the seven dimensions of the indicator. Additionally, these results also provide information on the variables that best adapt to each magnitude of decent work (Kahn, 1990) [99].

**Table 1:** Variable’s description.

JQI	ITEMS
Physical environment (JQI Physical)	Posture-related (ergonomic) risks
	Ambient risks
	Chemical risks
	Biological risk
Work intensity (JQI Intensity)	Quantitative demands
	Pace determinants and interdependency
	Emotional demands
Working time quality (JQI Working time)	Duration
	Atypical working time
	Working time arrangements
	Flexibility
Social environment (JQI Social)	Social behavior
	Social support
Prospects (JQI Prospects)	Employment status
	Career prospects
	Job Security
	Downsizing
Skills and discretion (JQI Skills)	Cognitive Dimension
	Decision latitude
	Organizational participation
	Training

**Fig 1:**
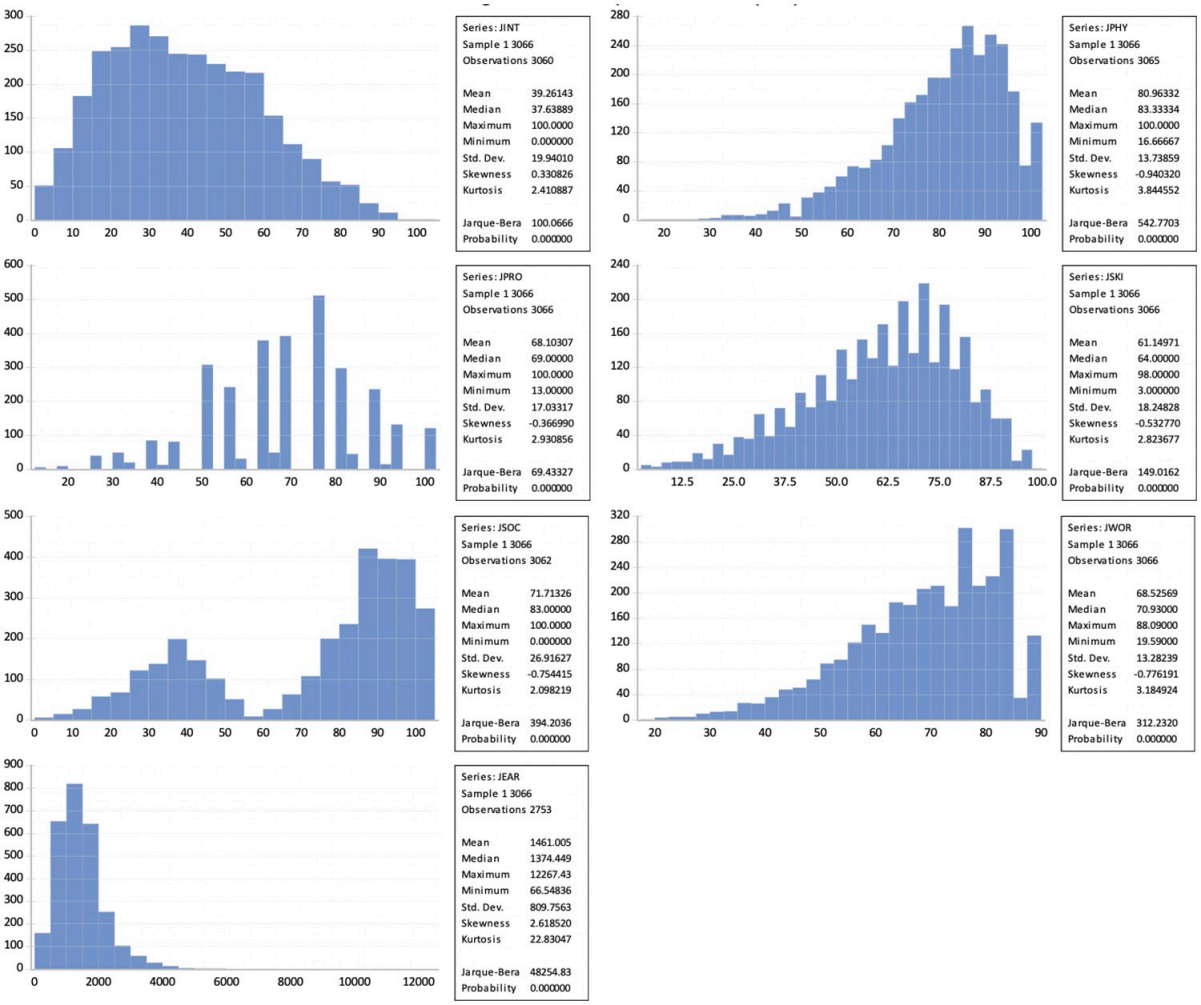
Description of factor (JQI).

Fig 1 show the distribution of the explanatory variables, as well as a brief descriptive summary of their descriptive measures. The different scales are made up of specific questions that allow obtaining a score on the nature of the work carried out by the respondents.

**Fig 2:**
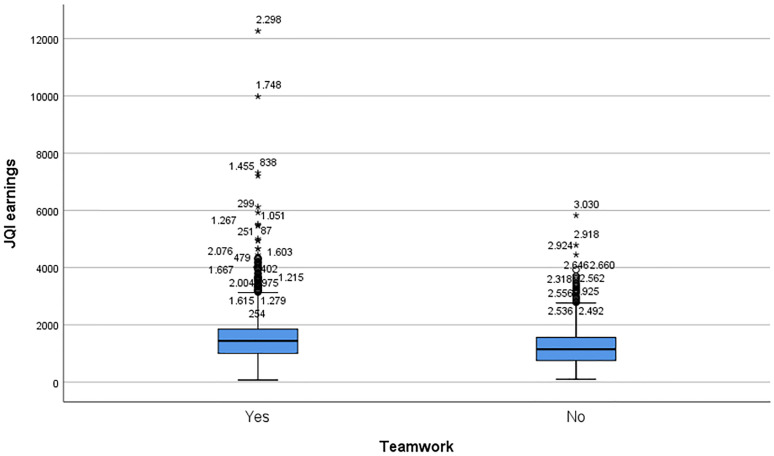
Distribution of JQIEarnings in each class defined by teamwork.

The last causal variable considered for the relevance of the study is teamwork. This variable takes the value 1 for those subjects who manifest this situation, compared to the value 2 in case of not working in a team. In the analysis of the groups of individuals who work or not in a team (Teamwork), it is observed that clear differences appear for the variables analyzed (Fig 2).

### 4.3 Method of analysis

**Fig 3:**
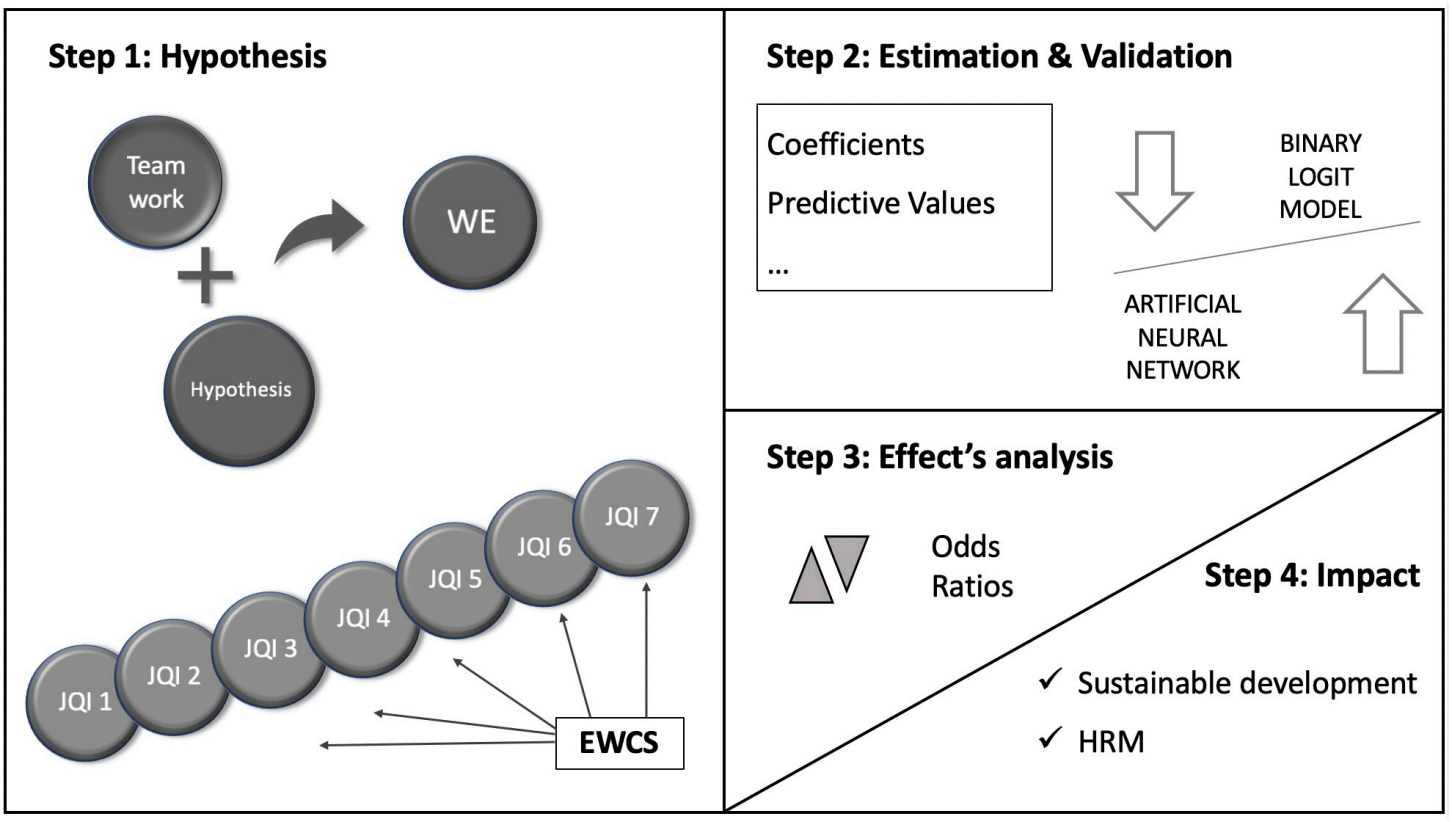
Phases of the research methodology.

A two-phase analysis has been carried out in order to test the research hypotheses proposed, and therefore to determine the relationships between work engagement (WE) and the factors associated with decent work (Fig 3).

To do this, several statistical techniques are employed. A binary logistic estimation is specified [100]. Subsequently, and in order to carry out the analysis of the hypotheses, we proceed to estimate an artificial neural network with the same exogenous variables; the results corroborate the effect of the causal variables proposed on the determination of the employees’ work engagement. The later procedures are non-linear models in which the relationship between the variables is established through a flexible structure and in which the information is transmitted from the exogenous or explanatory variables to the target endogenous variable through unobservable (latent) variables, called, for the ANNs, neurons, organized in layers, which receive input information, process it and transmit it to other variables in the next layer, until the output (WE) variable is reached. Finally, the coefficients obtained in both methods are evaluated in order to determine the effects on the work engagement of the characteristics of their work Table 2.

**Table 2:** Logit model.

*Dependent Variable: WE*				
Variable	Coefficient	Std. Error	Z-statistic	*p*-value
C	-0.682782	0.233021	-2.930130	0.0034
TEAMWORK	0.296658	0.092555	3.205192	0.0013
JQI PROSPECT	0.011012	0.002358	4.670457	0.0000
JQI INTENSITY × JQI EARNING	2.58E-06	8.86E-07	2.915974	0.0035
Akaike info criterion	1.349057	Restr. Deviance		3718.391
Schwarz criterion	1.359945	LR statistic		69.74887
Hannan-Quinn criter.	1.352993	P(LR statistic)		0.000000
Obs with *WE* = 0	1189	Total obs		2712
Obs with *WE* = 1	1523			

The estimated model is

$$WE=\frac{1}{1+e^{-u}}+error$$

All the explanatory variables are significant. The sign of the coefficients are in consonance with the research hypotheses about the direction of the influence of each exogenous variable on WE. The likelihood ratio statistic, LR = 69.75, shows a good overall fit. In relation to the predictive capacity of the model, for a probability cut-point, c = 0.565 used to balance the predictions in both groups, slightly more than 57% of correct predictions are obtained (Table 3).

**Table 3:** Logit model: Predicted-observed classifications.

Classification	*WE* = 0	*WE* = 1	Total
*P*(*WE* = 1)≤0.565	677	651	1328
*P*(*WE* = 1)>0.565	512	872	1384
Total	1189	1523	2712
Correct	677	872	1549
% Correct	56.94	57.26	57.12

**Fig 4:**
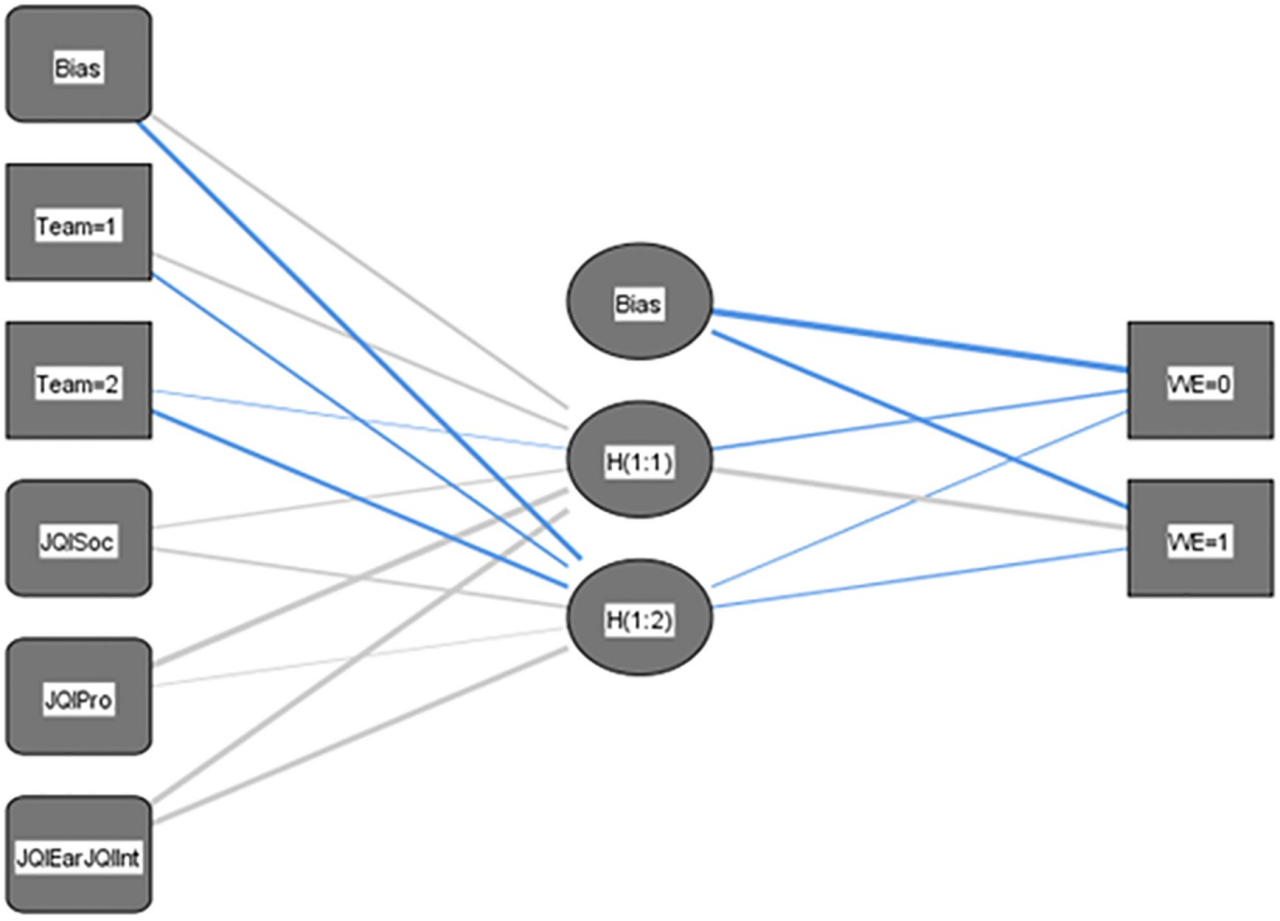
Artificial neural network MLP (5+1; 2; 2).

As indicated above, an alternative is to estimate a neural network to predict WE from the same variables used in the logistic regression, in order to corroborate the results presented with this model. A multilayer perceptron-type neural network is designed, configured by four input variables (JQI Factors) and TeamWork, a hidden layer with two neurons. The activation function used will be hyperbolic tangent (Fig 4).

The classification Table 4 is obtained, and it shows that it slightly improves the predictive capacity of the logit model, but the difference is not considered sufficient to conclude that ANN is preferable. Classification results with the network (Table 4) are obtained in a similar way as in logit models (Table 3). In this case, it is observed that the predictive power increases by 2%, although the neural network model with more degrees of freedom is considered preferable, since it estimated are considered more robust for prediction.

**Table 4:** ARN: Predicted-observed classifications.

Classification	*WE* = 0	*WE* = 1	Total
*P*(*WE* = 1)≤0.58	677	565	1242
*P*(*WE* = 1)>0.58	558	954	1512
Total	1235	1519	2754
Correct	677	954	1631
% Correct	54.82	62.80	59.22

## 5 Results

The relationship between HRM and WE through teamwork is expected because decent work is a measure of how working conditions meet multiple human needs. Additionally, considering the possibility of working as a team or not, it will be possible to draw conclusions about how HRM, through the dimensions of decent and sustainable work, identified by the SDGs impact on the WE in each group of workers. A logistic regression model was carried out incorporating as finally significant predictor variables, *JQI Prospects*(*b*_1_ = 0.011), *JQI Social Environment*(*b*_2_ = 0.005) and *JQI Work interaction intensity* × *JQI Earning* (*b*_3_ = 0.000) and *Teamwork* (*b*_4_ = −0.2966).

Therefore, these dimensions are configured as influencing work in all occupational categories, but each one with a particular relative importance. In the case of Teamwork, it is observed that the coefficient associated with the variable is negative, which indicates that not working in a team decreases the probability of improving work engagement, keeping the rest of the factors constant. It is presented as the main determinant of work engagement above the values of the JQI indices. Thus, analyzing the associated odds (0.743), it is observed how the fact of not working as a team reduces the work engagement of any worker by almost 25%, regardless of the assessment of the other JQI factors. This leads to reflect on these types of occupations where factors such as promotion, social environment, work intensity itself or even salary, although important for the individual, do not present a level of relative importance on the work engagement that provided by the interaction of his teammates. To a lesser extent, and in relation to the JQI prospects and JQI social environment variables were presented as the main determinants of WE, increasing the probability of work engagement as more beneficial are each of the factors.

The odds ratios obtained in order to evaluate the influence of each exogenous variables upon the WE are calculatesd. JQI Prospect, whose odds ratio is 1.011, it is concluded that the probability associated with a worker being committed to the organization (versus not being) increases by more than 10% with an increase of 10 percentage points in the assessment of the level of promotion of the position he occupies.

## 6 Discussion

The cooperation between all interested parties is key to the success of an organization, as these agents are involved with institutions and organizations complying with social expectations and following principles of responsible behavior [9]. Considering the growing demand for socially responsible behavior, the implementation of CSR practices has been established as a source of legitimacy [101]. Most of the research has focused on the external context of policy implementation [25], while from the internal perspective the impact of these policies remains relatively unexplored.

This work aims to identify how a work practice based on HRM, in the health sector, and as teamwork, can lead tot a sustainable and decent work scenario in the long term, achieving work engagement necessary to reach efficient levels of productivity. Following this idea, this objective is quantified on how the different dimensions of the Work Quality Index (JQI), created by Eurofound, influence the variations in work engagement. The proposed results reveal that teamwork is essential for work engagement from the perspective of sustainability in human resource management. These results are in accordance with the widely accepted point of view, in the academic literature, on the existence of different human resource management models that influence work engagement such as stated by [102]. However, there are no studies that indicate that teamwork influences work engagement and that analyze the factor with greater impact on work engagement, and, therefore the sustainability of these practices in the health sector [102]. In addition, this article pretends to develop a theoretical contribution by suggesting that teamwork as a mediating variable of work engagement in this sector in Europe, and also, to deepens the analysis of sustainability in human resource management. For this purpose, a logistic regression model is estimated linking the components of the HRM with the construct of work engagement through teamwork, and the influences of work dimensions on work engagement.

The models specified show which factors do influence the sustainability of the HRM of companies in terms of work engagement. Upon further study of these differences, it can be observed that work engagement through teamwork in the health sector can be explained using the variables prospects, social environment, intensity and earnings. Therefore, health sector managers should develop human resource strategies to improve employees’ engagement by acting on these variables. HRM is related through teamwork with prospects [103], social environment [104], intensity [105] and earnings [106] which reveals that teamwork originates a high engagement to sustainability in the economic and social dimensions of work.

Teamwork is fundamental in its relationship with WE, because the work dimensions that most affect depend directly on the discretion of the team’s operation-coercive pressures. The results show that the dimensions related to HRM that most influence work engagement are prospects [107], social environment [108], intensity [109], and earnings [110]. Regarding the personnel policy related to teamwork, there are dimensions of the HRM related to the functional approach of [111], which are those related to performance and those in charge of maintaining the equipment. In work team environments, the performance function implies the achievement of team objectives related to work (prospects, intensity and earnings) and are related to its regulation. The maintenance function involves keeping team members together (social environment). The prospects involve how the work team guides the work actions to achieve the fulfillment of the main objectives, with the focus on the analysis and planning of the activity. Team members must understand the team’s goals and have a shared vision for them. This is especially important for team members who have never worked together before [112]. When these members analyze the team’s purpose in the organization, they seek and process information about the objectives that they must achieve and the conditions to proceed. In addition, they determine their preferences and abilities to find out what contribution each team member can make to the desired objectives.

Regarding the social environment, some personal or interpersonal relationship problems can damage team maintenance; for example, personal difficulties faced by team members and conflicts between team members [113]. Additionally, these difficulties can prevent team members from making full contributions to complete team tasks or performance. When team members encounter personal difficulties, such as failures, temporary stress, and safety issues at work, their colleagues can provide active support to help them overcome these difficulties [79]. This dimension is linked to motivating or building trust [114], team spirit and morals [115] or personal and social support [116]. Intensity, the collective nature of team tasks, means that team members interact and share resources to complete their objectives, that is, they are interdependent in accomplishing tasks [117]. Additionally, individual efforts must be aligned and coordinated by keeping team members together [118]. Finally, in relation to earnings, incentives can be provided to team members to perform better, and to maintain high levels of performance [119].

These conclusions seem to indicate the usefulness of teamwork to explain the differences in human resource management in the labor dimensions that therefore influence sustainability. The success of any organization cannot be based solely on the factors mentioned above, but must pay attention to the organizational perspective and specifically, to the policy of responsible management of human resources. Teamwork coupled with work engagement are key to good organizational functioning and business performance [41]. The importance of teamwork to improve work engagement and performance was already pointed out [120, 121], and, recently, [122]. As expected, these results were also observed in the healthcare sector [123]. In this way, the health sector could find that work intensity is a positive stressor that reinforces engagement if they are able to work as a team, challenging their abilities and being fairly recognized by the organization [124].

## 7 Conclusions

This article comparatively analyzes the levels of sustainability in human resource management through the mediation of teamwork in work engagement. The results show that labor dimensions most influence work engagement of a sustainable human resources management system. The study analyzes which job dimensions that can be modified to adopt a sustainable human resource management system. Differences in work factors may also explain aggregate differences in work engagement derived from human resource management.

### 7.1 Implications for theory

This research contributes to the current literature in several ways. The theoretical implications provided by this study are based on the use of teamwork as a mediating variable in the sustainability of HRM, as this work is one of the first attempts to systematically compare the sustainable human resource management of companies in the health sector, following [125], and it could strengthen the literature on sustainable human resource management. The mediation of teamwork in work engagement can help to interpret differences in the sustainability of human resource management. A sustainable human resource management system by companies, an aspect that the literature has barely investigated [126]. The proposed results suggest that teamwork in the management of sustainable human resources causes more work engagement than if it is not used. In addition, the labor dimensions that most influence labor engagement are prospects, social environment, intensity and earnings. Therefore, the internal management of work teams seems to influence the sustainability of the health sector in Europe.

### 7.2 Practical implications

Regarding the practical implications, the results indicate that the human resource management models established in the health sector do not adequately reflect the sustainability required at the normative and legal level by the ODS. Previous studies that used sustainability in the management of human resources in the health environment, such as the [127] study where it analyzes the sustainability of the sector in theoretical terms; or as in the study by [128], where they carry out a systematic review that integrates empirical research on teamwork, where most of them were based on quantitative methods, analyzing interpersonal processes, transition processes, and processes of action. This study clarifies that healthcare workers feel good when working as a team. In addition, this research also proposes to the healthcare community that human resource professionals can use the tools at their disposal to propose effective human resource strategies to improve personal well-being. The health sector is organized around teamwork. Teamwork is a type of human resource management that indexes the work engagement of workers, a key aspect for companies, because it is a determining factor in long-term organizational success. Teamwork greatly influences the level of engagement of employees to their work, as it leads to a positive, high-energy affective-motivational state combined with high levels of dedication and a strong focus on work [129].

### 7.3 Health policy recommendations

With the aim of establishing forms of business management that increase work engagement, a new organizational trend has emerged that may be the key to a job change in terms of sustainability. On the one hand, the market has favored the increase in techniques based on personnel management, which poses a new organizational paradigm through innovative forms of business management. Teamwork in terms of time horizon, autonomy and remuneration has become more favorable for workers according to their professional category in the health sector. As a consequence, a scenario is observed that can make a sustainable difference as a norm in the European context. Given that teamwork influences work engagement, it is expected that this organizational system will present differences in the labor sustainability of the sector, widening this gap based on determining factors for teamwork. As shown in our empirical results, the most effective strategy in terms of work quality to improve work engagement through teamwork is the development of resource policies that promote the professional career, social environment, intensity and earnings with optimal training courses. training to improve the skills of healthcare workers, as well as offering them clear career prospects.

### 7.4 Limitations

Despite the advantages of using ECWS data, they present some limitations that affect some methodological aspects. First, the measures in this database are self-reported, so they can present a certain bias motivated by the implicit social desirability that some questions provoke. This bias can be especially important in the case of wages, work history, type of contract, social relationships with colleagues and or supervisors, as well as other sensitive issues in the work environment that may suffer conscious or unconscious biases caused by perception of workers than they “would like it to be” compared to the country’s standard of living, economic conditions, and so on. Second, despite including a large battery of variables, the study is cross-sectional in nature and was subject to the limitations that this entails.
